# The COVID-19 pandemic and economic recovery: The mediating role of governance, a global perspective

**DOI:** 10.1016/j.heliyon.2024.e39869

**Published:** 2024-10-28

**Authors:** Regret Sunge, Calvin Mudzingiri, Nkosingiphile Mkhize

**Affiliations:** a University of the Free State, Afromontane Research Unit, Faculty of Economic and Management Sciences, Department of Economics and Finance, South Africa; b University of the Free State, Faculty of Economic and Management Sciences, Department of Public Administration and Management, South Africa

**Keywords:** COVID-19 pandemic, Governance, Economic recovery, Mediation analysis, Structural equation modelling

## Abstract

The COVID-19 pandemic, arguably the most extensive economic shock after the Great Depression, has drawn attention from policy custodians over the past three years. Governments’ response brought to the limelight the role that governance plays in mitigating the economic shrinking effects of a pandemic. This study investigated the mediating role of governance in the post-pandemic recovery process using structural equation modelling of cross-sectional data from 125 countries for the years 2020 and 2021. The results show that governance did not mediate economic recovery at the global level. However, regional analysis reveals a full mediation effect in Africa and for low-income countries in 2021. Disaggregating governance by indicators in Africa reveals complete mediation for control of corruption, government effectiveness, regulatory quality, and the rule of law. Achieving sustainable economic recovery requires strengthening local governance structures and encouraging international cooperation. The research motivates the establishment of international governance institutions in the spirit of United Nations-driven frameworks. This can be complemented by country-specific, multi-agency, cross-sector collaborations led by the state, the development of governance systems that reduce mistrust among stockholders, and investment in artificial intelligence and e-governance systems.

## 1 Introduction and contextual background

No event has been more detrimental to global economic growth and sustainable development in the 21st century than the COVID-19 pandemic. The outbreak brought a shock that transmitted through all sectors of the economy. In 2020, the global economy registered negative growth of 3 % against the backdrop of negative growth in sectoral value-added output in manufacturing (−4.2 %) and services (−3.3 %) [1]. In international terms, trade in goods and services plunged by 12 % [2], while international tourism fell by an unprecedented 74 % [3]. Consequently, the world experienced a spike in poverty and food insecurity. The United Nations (UN) reported that the pandemic forced 119 to 124 million people into poverty and chronic hunger; accordingly, measures needed to be taken for the economy to recover from the COVID-19-induced regression [4]. Indeed, the global economy recovered; registering 6.02 % growth in 2021. Economic recovery depicts a period of economic expansion following a downturn or recession [5]. Economic recovery is usually proxied by growth in gross domestic product (GDP), which is a measure adopted in this study. Although the world economy recovered, little is known about the factors that contributed to the resuscitation.

While many factors can be linked to the economy’s recovery from the COVID-19 pandemic, this study examined the mediating role of governance. The research hypothesised that in times of crisis, good public governance matters more than ever [6]. The rationale for focusing on governance is an acknowledgement that most measures taken to mitigate the effects of COVID-19 involved public administration. This involved legal, regulatory, political, and safety net decisions where governments were critical in policy formulation, implementation, and action. According to the International Monetary Fund, a nation’s governance policies, institutions, and decision-making processes all contribute significantly to countries’ economic and social climate response to the pandemic [7]. This thinking is premised on the new institutional economics theory in which economic institutions, led by governments, are rational and altruistic agents that are incentivised to bargain politically for mutual gains [[8], [9], [10]]. Resultantly, good institutions reduce the cost of operating an economic system, allocate resources efficiently, and therefore produce socially efficient outcomes [11]. Governance is synonymous with institutional quality and refers to the way power is exercised in managing a country’s economic and social resources [12]. In this study, governance was measured using six Worldwide Governance Indicators^1^ computed by the World Bank [13].

The COVID-19 pandemic is not the last crisis the world will experience. With increasing uncertainties fuelled by tightening geo-political tensions and increasing climate change effects, it is a matter of when and not whether the next shock will be experienced. In this regard, the world should be better prepared to deal with abrupt economic shocks. Understanding the role of governance in the recovery process is therefore critical. Good governance should be associated with better preparedness and response, and reduced economic impact in case of shocks. Nonetheless, developments in the aftermath of the pandemic unearthed doubts about governance’s role in such circumstances.

**Fig. 1:**
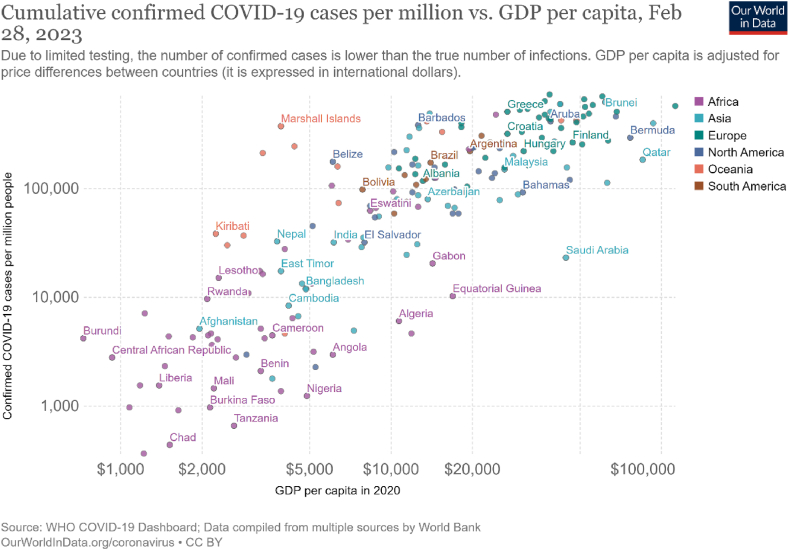
Cumulative confirmed COVID-19 cases per million vs. GDP per capita [16].

Data from the World Bank [14] showed that regions with higher income per capita were the hardest hit by the pandemic (see Fig. 1). The United Kingdom (UK) (Europe) and Chad (Africa) are a case in point. The UK, with a GDP per capita of US$42 676.00 (2020 international prices), recorded cumulative confirmed COVID-19 cases of 361 290.3 per million as of February 2023. Over comparable dates, Chad had a GDP per capita of just US$1519.00 while recording cumulative confirmed cases of 433.21 per million. In terms of economic shock, in 2020, the UK’s economy registered −11.03 % growth, while Chad recorded a modest decline of −1.6 % [1]. Ironically, the UK had superior governance compared to Chad. In 2020, based on the World Bank’s Worldwide Governance Indicators, the average governance value for the UK was 1.3 compared to Chad (−1.35) [15]. In 2021, the governance score deteriorated by 0.8 and 0.27 units in the UK and Chad respectively. This highlights that more developed and better-governed countries were the most affected by the pandemic. However, the UK’s recovery was more robust; registering 7 % growth in 2021, while Chad’s growth remained negative (−1.2 %), which indicates that better governance could have played a vital role in the recovery process.

Given this background, the broad objective of this study was to examine whether, how, and to what extent governance mediated economic recovery after the COVID-19 epidemic. The specific objective was to assess how the mediation role varied with regions and income levels. In doing so, the study makes two contributions to literature.

The first novelty is to elaborate on the mediating role of governance in economic recovery from COVID-19. Some studies have examined the impact of governance on economic growth [[16], [17], [18], [19]]. Despite using data points beyond 2019, the effect of the COVID-19 pandemic was not examined directly by these studies. Another class of studies investigated the effect of COVID-19 on economic growth [[20], [21], [22], [23]]. While significant findings were documented, the critical role of governance was not accounted for. Available evidence on the impact of COVID-19 on governance [[24], [25], [26]] did not assess the economic effects. By examining the mediating role of governance in the COVID-19 economic growth nexus, this study provides the first evidence that connects the three. It is logical that governance did not directly affect growth. Instead, it acted as a catalyst that supported growth enablers and as a neutraliser to growth detractors. Following the outbreak of the COVID-19 pandemic, the study posits that governance played the latter role.

The study employed mediation analysis using partial least squares structural equation modelling (PLS-SEM) estimation to achieve this. Previous studies corroborated the role of governance in economic growth [27,28]. Secondly, a global study provides a new dimension by further disaggregating the mediating role of governance by region and income levels. Such an analysis allowed the authors to control for regional and income-level idiosyncratic factors that shape the mediating role of governance in economic growth in times of crises. Conducting such an analysis is logical in crafting governance policies that answers to the heterogeneity in different regions.

The findings of this study are expected to cast light on the crucial role that governance plays in shaping the economic landscape post-pandemic. Policymakers can develop evidence-based strategies to foster more resilient and sustainable economic recovery trajectories by grasping the function of governance as a mediator. In addition, this research contributes to the growing body of knowledge on crisis management, public policy, and the complexities of governance in times of unprecedented challenges; ultimately yielding valuable insights for constructing more resilient and adaptable economies in the face of potential crises.

The study is organised as follows: Section 2 discusses theoretical and empirical literature, Section 3 details the study’s methodology and data analysis, the results are presented and discussed in Section 4, while the study’s conclusions and recommendations are provided in Section 5.

## 2 Literature review

This section reviews theoretical literature connecting governance to economic growth and empirical evidence related to COVID-19, governance, and economic growth (recovery). The review identified three literature thematics: (1) governance and COVID-19, (2) economic growth and COVID-19, and (3) governance and economic growth.

### 2.1 Theoretical literature

The relevance of governance in the economy emanates from the old right-wing-left-wing debate on economic management. Rightists or Neoclassicals believe in free market systems, while leftists or Keynesians advocate for market control and state intervention [29]. The former posit that economic recovery is stimulated by natural self-correction of the supply and demand mechanisms, flexible wages and prices, technological innovation, labour productivity, and capital accumulation. The role of governance in economic recovery therefore belongs to the latter. This study argues that strong institutions assisted economic recovery post-COVID-19. Specifically, the transmission mechanism is rooted in institutional economic thinking as established by Refs. [8,9,11,30,31]. Basically, the theory subscribes that growth is not a purely economic process and argues that political/institutional factors are decisive.

The importance of governance in growth was made popular by the new institutional economics theory [8,11]. The theory emerged from the explanation of the demographic, price, and productivity behaviour in Western Europe that defied Malthusian predictions. In a Malthusian framework, following periods of increasing production and prosperity, population growth triggers diminishing returns to production, which cause real wages and living standards to fall [32]. Nonetheless, in Western Europe, sustained increase in productivity outpaced diminishing returns to the extent that output grew more rapidly than population. According to North and Thomas, the anomaly was explained by the theory of institutional change. To them, economic institutions, particularly property rights, direct economic agents’ behaviour, thereby determining whether the ultimate result is growth, stagnation, or decay [33]. This is supported by Refs. [9,31], who argued that two factors, namely (1) institutions and policy, and, less importantly, (2) culture, are primarily responsible for economic growth [34]. The logic behind this is that governments act as rational actors that are incentivised to bargain politically for mutual gains. Resultantly, democratically elected governments reduce the cost of operating an economic system and therefore produce socially efficient outcomes [35].

The disaggregation of transaction costs by Ref. [11] suits the COVID-19 recovery path. The costs can be costs of externalities, information asymmetry, and/or risk. The invention and production of COVID-19 vaccines were associated with positive and negative externalities that warranted government subsidisation or internalisation measures. This safeguarded profits from the development of vaccines by private health scientists and sped up the rate at which vaccines were developed and quickened the return to economic normalcy. Government systems also reduced the cost of information asymmetry to the benefit of health entities, which provided services to fight the pandemic. Furthermore, institutions spread risks across economic agents [10], thereby supporting health entrepreneurs to realise a higher return. Accordingly, good institutional arrangements improved the efficiency of the economy [8], which positively contributed to the fight against the pandemic. According to this view, countries with better governance were expected to recover faster than otherwise.

[30] provided a different dimension through which governance can stimulate economic recovery following some disturbance. He posits that governance influences growth by resolving resource allocation conflicts following some shocks. When the pre-existing relative positions of different economic groups are disturbed, conflicts regarding the allocation of resources often arise. A sovereign administration with ideal institutional and ideological frameworks can solve resource conflicts at lower costs. The COVID-19 pandemic disturbed the usual economic norm, and governments had to divert budgets from other sectors such as infrastructure to the health sector, which created resource conflicts. Governments with better institutions were therefore expected to reduce the opportunity cost of the resource reallocation, thereby contributing to the recovery process.

### 2.2 Empirical literature

#### 2.2.1 Governance and COVID-19

Studies in this category can be classified into two groups. The first group examined how governance affected the success of intervention strategies. For instance Ref. [36], used data from the World Bank Development Governance Indicators to investigate how institutional quality impacted vaccination distribution and uptake in 172 countries. K-Means, principal component analysis, and XGBoost methodologies were combined to conduct analysis. They found that vaccination success increased with better governance, with regulatory quality being the most important indicator. [37] disaggregated governance at national and sub-national levels, covering 86 countries across the globe. The results indicated that all forms of institutional quality stimulated vaccination uptake, with only democratisation being insignificant. The findings were maintained at the regional level, where governance was found to cause an even higher vaccination success rate. Similar results were documented by Ref. [38], who used VOSviewer econometric visualisations to show that better institutions were pivotal in China. From a policy perspective [39], assessed how governance affected COVID-19 stringency. A composite governance index was constructed from six World Bank Governance Indicators, and panel models were applied to data for 163 countries for 339 days. The results indicated that the level of stringency varied with governance levels. Specifically, it was proved that policy stringency was high in countries with medium governance levels. The findings validated earlier evidence by Ref. [40].

The other group centred on the impact of governance on COVID-19 morbidity and mortality. In this regard, [24]used the six World Bank Governance Indicator data on 198 countries. The countries were grouped into three clusters: politically stable, less corrupt, and more corrupt. Ironically, while recording more tests and vaccinations, stable countries reported more cases and fatalities per million than corrupt ones. The authors attributed the anomaly to poor COVID-19 data reporting. Whichever way, the results of this study reveal that the impact of governance is conditional on its level. In another study [25], reported negative effects of governance on cases and deaths after adjusting for non-governance variables in a negative binomial model. Their finding implied that better governance reduced morbidity and mortality. However, governance was found to be insignificant without controlling for non-governance variables. Accordingly, the findings indicated that while governance was vital, it was not a sufficient condition to lower morbidity and mortality. While evidence on the impact of governance on COVID-19 policy, morbidity, and mortality is available, contrasting evidence could not found. This is despite the possibility that the pandemic could have imposed pressure on governance as governments led the response [41].

#### 2.2.2 Economic growth and COVID-19

[42] linked diseases to economic growth through the Solow growth model anchored on population growth and a savings rate augmented by individual health wellbeing by inserting the model into two disease-spreading susceptible-infected-susceptible and susceptible-infected-recovered models. The results indicated that the pandemic steady-state per capita income was lower, which implies recessionary effects. As expected, evidence of the impact of COVID-19 on economic growth mirrors that of other crises, such as the Great Depression. For instance Ref. [43], employed mixed data sampling (MIDAS) and Unrestricted-MIDAS models on first-quarter 2020 data to forecast the impact of the COVID-19 pandemic on growth. They forecasted that the pandemic would significantly affect the economic growth trend in the United States of America (USA) and G7 countries. The recession was mainly anticipated based on increased uncertainty and decreased investment growth. The results led them to conclude that the recession impact was like that of the Great Depression. Similar evidence was provided by Ref. [44], who employed a spatial computable general equilibrium model to investigate the economic effect of COVID-19 in South Korea. The study found that a decrease in social interaction of 10 % resulting from lockdown measures could reduce the national GDP by 0.815 %–0.864 %.

[45] showed that COVID-19 caused the global recession by dampening global real estate capital flows in 2020; however, they noted a remarkable recovery in capital flows in 2021 as the COVID-19 pandemic subdued. [46] analysed the impact of the COVID-19 pandemic on the job market. Considering the decreased number of job advertisements, it was concluded that the pandemic significantly impacted job creation, wage income, and economic growth. [47] also related the effects of COVID-19 on the economy to three significant channels: health, trade, and financing [26]. investigated how the COVID-19 response affected economic growth by easing lockdowns, health improvements, and supply chain benefit effects of vaccination distribution. According to Ref. [26], the optimal COVID-19 vaccine distribution scenarios might have increased global economic benefits by 11.7 %.

#### 2.2.3 Governance and economic growth

This strand of evidence examined how governance related to economic growth from 2020. For instance Ref. [27], used data spanning 1996 to 2020 to investigate the mediating role of governance on the relationship between public debt and economic development in Pakistan. The cointegration of the variables was validated by the autoregressive distributed lag and error correction model techniques. According to Ref. [27], better institutional quality mitigated the negative impact of public debt on economic growth in Pakistan [48]. also confirmed that governance plays a role in promoting growth. The finding was based on panel and time series estimations for the world’s 10 largest economies. A similar result was confirmed in developed and emerging economies [49] and in developing countries, including in Africa [50,51].

Furthermore [28], reiterated that governance fuelled economic growth in 79 countries from 2005 to 2022. Another dimension was provided by Ref. [52], who used data for G7 countries from 1990 to 2020 to show that ecological governance promoted green growth. While the studies in this category indicated the importance of governance in economic growth, they could not incorporate the inevitable effects of COVID-19. This is despite the documented effects of governance on COVID-19 [[53], [54], [55], [56]] and the likely effect of COVID-19 on governance [57]. The hypothesis of this study is that an analysis of the governance-growth nexus post-2020 is incomplete if the COVID-19 pandemic is not accounted for.

#### 2.2.4 COVID-19, governance, and economic growth

The literature review showed that very few studies on the growth effects of COVID-19 bothered to recognise the role of governance. A post-COVID-19 study by Ref. [58] investigated the effects of six World Bank Governance Indicators on economic growth in Pakistan. While governance indicators were found to promote growth, the study did not examine how governance moderated the impact of COVID-19 on economic growth. Likewise [59], gathered perceptions of the effect of governance on Latvia’s recovery from the pandemic. However, how governance reduced the growth effects of COVID-19 could not be established. A close study was provided by Ref. [60]. The study examined the independent impact of governance and COVID-19 on economic growth in the Caribbean using random effects panel data estimation. Four governance indicators (voice and accountability, the rule of law, political stability and absence of violence, and government effectiveness) strengthened growth. However, control of corruption and regulatory quality were growth retarding. COVID-19 variables such as cases and deaths also negatively impacted the economy. The study could not capture the mediating role of governance, which remains an outstanding empirical question. The next section details this study’s methodology used to analyse the data.

## 3 Methodology

### 3.1 Mediation analysis

We used mediation analysis to examine the role of governance in economic recovery from COVID-19. Mediation analysis is an approach to indirectly examine a proposed cause’s effect on some outcome through a suggested mediator [61]. The choice of mediation analysis was motivated by its advantage over conventional direct regression analysis. It provides a more functional understanding of how the predictor variable affects the predicted variable [62]. Accordingly, it allows researchers to capture the role of those variables that indirectly affect the outcome variable. Usually, such variables are neglected in traditional regression models. Mediation exists when a variable, the mediator (M), intercedes between an independent/exogenous/predictor variable (X) and a dependent/endogenous/predicted variable (Y) [63]. Specifically, a change in X causes a change in M, which, in turn, transmits the effect to Y [64]. Following the literature that identified governance as a driver of economic growth [[16], [17], [18]], it was considered a mediator in economic recovery.

**Fig. 2:**
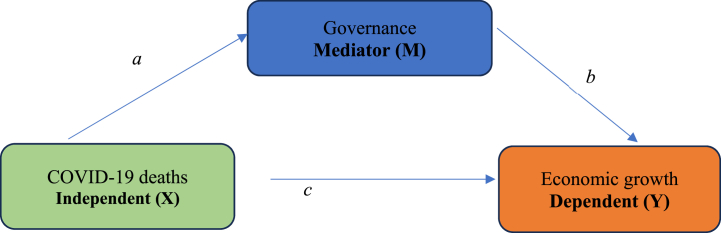
Testing for mediation: The Baron and Kenny (BK) approach.

In the mediation analysis, the effect of X (COVID-19 deaths) on Y (economic growth) was decomposed into three: direct, indirect, and total effect, as shown in Fig. 2. Direct effect (c) exists when the effect of X on Y is not influenced by M (governance). Indirect effect (a∗b) occurs when the effect of X on Y is influenced or mediated by M. The total effect (a+b+c) is the sum of the direct and indirect effects. The pathway to mediation analysis is illustrated in Fig. 2.

Testing for mediation has attracted much debate, and more insights are emerging [65]. pioneered mediation validation through the causal step approach or the classical test. Their method, which follows hierarchical multiple regression, involved satisfying three conditions: (1) changes in X significantly cause changes in M, (2) changes in M significantly cause changes in Y, and (3) if paths a and b are controlled, path c, which was previously significant, becomes insignificant, with complete mediation occurring when c is zero. The hierarchical multiple regression can be expressed in three causal models, as shown in Equations [1], [2], [3]):

[1] $$Y=i_{1}+cX+\varepsilon_{1}$$

[2] $$M=i_{2}+aX+\varepsilon_{2}$$

[3] $$Y=i_{3}+bM+\varepsilon_{3}$$

Where $i_{i=1,2,3}$ and $\varepsilon_{i=1,2,3}$ are constants and error terms for the three models respectively. If at least one of a, b, and c is insignificant, there is no mediation; otherwise, one can proceed to test for mediation. This is done by regressing Y on both X and M to give Equation (4):

[4] $$Y=i_{4}+cX+bM+\varepsilon_{4}$$

Given Equation (4), if c is still significant but at a lower level and/or smaller size [66], then there is partial mediation [65]. augmented their approach with the Sobel (1982) z-test [67]. The Sobel test checks the statistical significance of the indirect effect. In principle, the Sobel test examines if including M significantly reduces the impact of X on Y [68]. Accordingly, the null hypothesis (H_o_) is that after controlling for M, the difference between c and a∗b is not statistically significant. Rejecting H_o_ therefore implies either total or partial mediation. The Sobel z-test scores are computed as shown in Equation (5):

[5] $$z=\frac{a*b}{\sqrt{b^{2}s_{a}^{2}+a^{2}s_{b}^{2}}}$$

Where $s_{a}^{2}$ and $s_{b}^{2}$ are squared standard errors for a and b, the BK and the Sobel z-test have become the standard instruments for testing for mediation. Nonetheless, several criticisms have been raised, which led to more acceptable approaches.

### 3.2 The Zhao, Lynch, and Chen (ZLC) (2010) approach

Perhaps the most notable arguments were given by Ref. [69], who raised three issues. Firstly, they registered discomfort with the claim in the BK approach that mediation is most robust in the presence of an indirect effect and the absence of a direct effect. Instead [69], advanced that the presence and depth of mediation should be deduced from the size of the indirect effect rather than the mere absence of the direct effect. Accordingly, the first step in the BK approach could be misleading as detecting indirect and direct effects is possible. Under the BK approach, this draws a line between partial and complete mediation. However, in practice, as [70] showed, it is possible to add more indirect effects if c is significant. Accordingly, the BK approach does not allow a complete examination of the nature of the mediation.

Secondly [69], posited that Stage 1 is not necessary. The most essential condition to be met is that the indirect effect a∗b should be significant. The other stages of the BK approach are only necessary in classifying the type of mediation.

Thirdly, the reliance on the Sobel test has been disproved based on its low power [69], inefficiency in small samples [70], and incapability to demonstrate that the effect of X on Y diminishes after adding M to the model [61]. To address the shortcomings of the BK approach [69], suggested three forms of mediation and two forms of non-mediation.

**Fig. 3:**
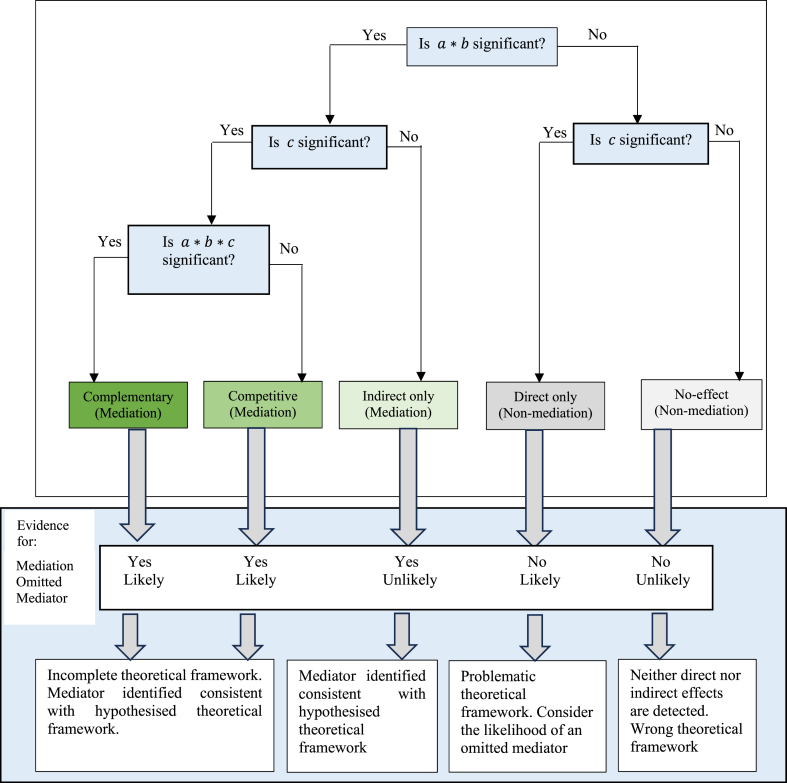
Decision tree for establishing and understanding types of mediation and non-mediation.^2^.

In the ZLC approach, mediation can take the form of (1) complementary, (2) competitive, and (3) indirect only [63]. In the first category, the indirect and direct effects are significant and go in the same direction. When mediation is competitive, both effects are significant but are antagonistic. For indirect-only mediation, only the indirect effect is significant. Hence, in addition to partial (complementary) and indirect-only (complete) [69], added competitive mediation, also termed the suppressor effect. The two types of non-mediation are classified as direct only and no effect. Only the direct effect is significant in the former, and both effects are insignificant in the latter. The template of the ZLC mediation approach is shown in Fig. 3.

The lower panel in Fig. 3 shows the presence or absence of the hypothesised mediators and the possibility of omitted mediators under each outcome. For all three forms of mediation, the outcome supports that M plays a mediating role. Only under complete mediation is when there is no case of omitted variables. For non-mediation, all scenarios do not support the hypothesised mediation. Lastly, omitted mediation is reflected under direct-only mediation.

Given the framework above, the approach by Ref. [69] was used to specify the mediation models as shown below:

[6] $${Ec\_growth}_{i1}=i_{i1}+clg{COVID\_19}_{i1}+\varepsilon_{i1}$$

[7] $${Governance}_{i2}=i_{i2}+alg{COVID\_19}_{i2}+\varepsilon_{i2}$$

[8] $${Ec\_growth}_{i3}=i_{i3}+b{Governance}_{i3}+\varepsilon_{i3}$$

[9] $${Ec\_growth}_{i4}=i_{i4}+clg{COVID\_19}_{i4}+b{Governance}_{i4}+\varepsilon_{i4}$$

Where Ec_growth is the dependent variable (Y) and measured by economic growth rate, COVID_19 is the independent variable (X) measured by COVID-19 deaths per million, in logarithm form (lg), and governance is the mediator variable (M) proxied by different governance indicators. The governance indicators used in this study are explained in Table 1. k=1,2...125 denotes the countries in the sample. To control for heterogeneity in governance’s mediating role, the data were further disaggregated into five regions: Africa, Asia, Europe (including Australia), North America, and South America. Countries in each region were included or excluded based on data availability for all the variables used in the analysis*.* To obtain coefficients a,b,andc, the maximum likelihood estimator (MLE) under the PLS-SEM approach was used.

**Table 1:** Data description and sources.

**Variable**		**Description**	**Source**
**Economic growth**		Annual percentage growth rate of GDP at market prices based on constant local currency. Aggregates are based on constant 2015 prices, expressed in United States dollars (USD).	[71]
**COVID-19**		Confirmed COVID-19-related deaths per million.	[16]
**Governance**	**Indicator**	Estimates of governance ranges from approximately −2.5 (weak) to 2.5 (strong).	
	Control of corruption	Captures perceptions of the extent to which public power is exercised for private gain and “state capture” by elites and private interests.	[72]
	Government effectiveness	Captures perceptions of the quality of public and civil services, its independence from political pressures, the quality of policy formulation and implementation, and the credibility of the government’s commitment to such policies.	
	Political stability and absence of violence	Measures perceptions of the likelihood of political instability and politically motivated violence, including terrorism.	
	Regulatory quality	Captures perceptions of the government’s ability to formulate and implement sound policies and regulations that permit and promote private sector development.	
	Rule of law	Captures perceptions of the extent to which agents have confidence in and abide by the rules of society, particularly the quality of contract enforcement, property rights, the police, and the courts, as well as the likelihood of crime and violence.	
	Voice and accountability	Captures perceptions of the extent to which a country’s citizens can participate in selecting their government, as well as freedom of expression, freedom of association, and free media.	
	Governance Index	An index of the six governance indicators.	Computed using principal component analysis^3^ from the six measures of governance above.

### 3.3 Partial least squares structural equation modelling (PLS-SEM)

Structural equation modelling (SEM) is the standard approach for executing mediation relationships. Several advantages substantiate the approach. Firstly, unlike [65], in which models were estimated in sequence, SEM estimates Models 1 to 4 simultaneously. Secondly, SEM can test causal relationships in complex relationships [62]. With regression models, exogenous and endogenous variables are specified. However, in the SEM approach, the endogenous variable in one model is transformed into the independent variable in the other model. Regardless, the SEM technique can handle the complexity of such specifications. Thirdly, SEM permits modelling the measurement error for dependent and independent variables. This provides high flexibility in estimating the correlation between error terms [73].

Two approaches, common factor-based (CB) SEM and PLS-SEM, are commonly used in analysing relationships in structural equation models. This study used the PLS-SEM approach, based on its superiority over the CB-SEM. They differ on the treatment of latent variables. In CB-SEM, constructs are designated as common factors that explain covariation between indicators [74]. In PLS-SEM, the common factor assumption is relaxed, and concepts of interest are treated as weighted composites. According to PLS, composite variables are deemed comprehensive representations of the constructs, thereby producing valid proxies of variables under examination. When using CB-SEM, researchers need to impose specific constraints on the model, which usually contradict theoretical underpinnings [75].

There are many estimators for SEM, such as maximum likelihood, generalised least squares, and weighted least squares. The MLE is the default estimator for SEM; hence its use in this study. The MLE assumes that (1) the joint distribution of the variables is not skewed, (2) the variables are continuous, and (3) there are few or no missing data points [76]. These conditions are in line with the data.

An important step was to check the model’s goodness of fit (GOF) to avoid Type I and Type II errors [77]. Traditionally, the loglikelihood ratio (LR) chi-square ($\chi^{2}$) test has been used to check GOF in SEMs. Typically, the GOF of a given model M is checked by the $\chi^{2}$ test statistic, T, defined as n times the size of the discrepancy function that examines the observed covariance matrix in the sample and the fitted covariance matrices based on parameter estimates and the given model [78]. Given the conventional assumption that M holds, T should follow an asymptotically $\chi^{2}$ distributed function. Hence, M is rejected if $T>C_{\alpha }$, where $C_{\alpha }$ is the critical value statistic. Accordingly, a significant p-value (p < 0.05) suggests that the model is unsuitable. However, the LR $\chi^{2}$ test is heavily criticised for its dependence on sample size. The dependence means that any minor misspecification is easily picked up by the $\chi^{2}$ test, which will lead to a Type I error [77,78]. This has led to the development of better GOF tests, particularly the baseline comparison (comparative fit index [CFI] and Tucker-Lewis index [TLI]) and the root mean squared error of approximation (RMSEA) tests.

The CFI measures the GOF of the hypothesised model versus the baseline model. Usually, the model with no restrictions on the variance with all covariances fixed to zero is treated as the baseline model. In Equation (10), Δ compares the non-centrality parameter in the hypothesised and baseline model [77]:

[10] $$\Delta =1-\frac{\lambda_{Hypothesised}}{\lambda_{Baseline}}$$

In Equation (10), the GOF depends on the fitness of the hypothesised model relative to the baseline. If the former is as poorly fit as the former, then Δ = 0 reflects complete unfitness. However, the better fit is the former to the latter, Δ∼1, which reflects increasing fitness. The TLI is the same as the CFI approach. The RMSEA test estimates the error of approximation of the model in the population separately from the error of estimation due to sampling error. Using this method, the specification is rejected when $FI>C_{fi}$ FI, where FI is the fitness index and $C_{fi}$ is the benchmark value for the given fitness index. The condition above implies a significant p-value (p < 0.05); hence, an insignificant statistic is ideal. When p > 0.05, the RMSEA will be small enough to warrant any model misfit. The literature considers an RMSEA <0.05 as a close fit [79].

### 3.4 Data description and sources

The study used data from 125 countries drawn from six regions for the years 2020 and 2021. The list of countries in each region is given in Appendix 1. The number of countries was restricted by missing and/or incomplete data. COVID-19 was first reported in December 2019, became a pandemic in 2020, and cooled off in 2021. Accordingly, the year 2019 could not be included because it was too late to cause meaningful disturbances in the economy. Beyond 2021, the effects of COVID-19 were secondary and indirect; hence the focus on 2020 and 2021. To compare the mediating role of governance in economic recovery, the study conducted cross-sectional data analysis for the two years. While cross-sectional analysis cannot capture time variations [80], it is ideal for studying event outcomes, particularly in understanding the prevalence of health outcomes [81]. Given that the pandemic was more of an event, waiting for time series observation would make the evidence absolute.

## 4 Results and discussion

This section presents, interprets, and discusses the descriptive statistics, MLE results, and the mediation tests results.

### 4.1 Descriptive statistics

The data in Table 2 show that in 2020, in all regions in the sample, economic growth plummeted to −3.92 %. The growth impact was the lowest in Africa (−1.72 %) and the highest in North America (−9.74 %). Countries in North America had the highest variability/standard deviation in economic growth (6.27 %), while South American countries had the least variation. Considering individual economy growth impact, the Bahamas in North America registered the biggest decline (−23.82 %), while Ireland was the most resilient and recorded economic growth of 6.18 %. In 2021, all regions recovered from the pandemic, with growth averaging 5.7 %. The biggest recovery was recorded in North America, whose economy increased by 17.69 percentage points from −9.74 % to 7.95 %. Turning to COVID-19 deaths, South America had the highest average cases per million 2020 (846) and 2021 (1649). At the country level, Peru (2 733) and Bulgaria (3 434) had the highest deaths per million in 2020 and 2021, respectively. With regard to governance, Africa had the most inferior score in 2020 (−0.68) and 2021 (−0.67). Despite the poor governance rating, the economic impact of COVID-19 was not the most pronounced in Africa. At the country level, the weakest governance was recorded in Somalia in 2020 (−2.07) and 2021 (−2.06).

**Table 2:** Descriptive statistics, 2020 and 2021.

Region	Variable	Obs	Mean		Std. Dev.		Min.		Max.	
			2020	2021	2020	2021	2020	2021	2020	2021
All regions	COVID deaths/million	125	346.53	740.98	440.47	785.54	0.155	0.64	2733.25	3433.52
	Economic growth	125	−3.92	5.376	5.07	4.28	−23.82	−17.91	6.19	15.34
	Governance	125	−0.01	−0.01	0.98	0.98	−2.07	−2.08	2.01	2.03
Africa	COVID deaths/million	40	51.13	213.26	97.96	400.44	0.155	0.931	468.04	1695.03
	Economic growth	40	−1.72	4.12	4.48	3.04	−14.60	−2.20	6.06	11.37
	Governance	40	−0.68	−0.67	0.67	0.67	−2.07	−2.06	0.89	0.89
Asia	COVID deaths/million	24	159.01	493.96	198.83	435.35	0.29	0.639	718.62	1716.53
	Economic growth	24	−3.84	3.467	5.52	5.64	−21.40	−17.91	3.45	8.68
	Governance	24	−0.18	−0.19	0.88	0.89	−1.66	−1.68	1.83	1.85
Europe	COVID deaths/million	38	613.76	1218.18	416.19	898.03	4.82	5.02	1687.90	3433.52
	Economic growth	38	−3.86	6.63	3.56	3.10	−11.33	2.3	6.19	13.95
	Governance	38	0.72	0.73	0.86	0.87	−1.09	−1.17	1.91	1.93
North America	COVID deaths/million	13	498.96	849.19	409.13	443.11	20.37	46.35	1157.79	1536.03
	Economic growth	13	−9.74	7.95	6.27	5.57	−23.82	−1.80	−1.76	15.34
	Governance	13	0.04	0.01	0.82	0.86	−1.27	−1.37	1.51	1.51
South America	COVID deaths/million	9	845.59	1649.92	763.85	741.37	49.08	857.73	2733.25	3216.43
	Economic growth	9	−6.15	7.41	2.99	3.60	−10.95	4.10	−0.82	13.35
	Governance	9	0.05	0.02	0.65	0.66	−0.75	−0.85	1.07	1.11

### 4.2 Maximum likelihood estimation (MLE) results

In line with the main objective, we analysed the mediation of governance in the COVID-19-economic growth relationship using cross-sectional data for the years 2020 and 2021 at three disaggregated levels: (1) global, (2) regional, and (3) different income levels. The results^4^ are presented in Table 3, Table 4, Table 5, Table 6. Each table has five panels labelled A to E, which show results for (A) the effect of COVID-19 on governance (a coefficient), (B) the effect of COVID-19 and governance on economic growth (coefficients b and c), (C) significance tests of the indirect effect, (D) for mediation tests, and (E) GOF tests. The central question this research sought to answer was whether governance played a mediating role in the COVID-19-economic growth relationship. The BK and the ZLC approaches were used, and in cases of conflict, the latter was preferred, given the advantages outlined in Section 3. In addition, the ratio of indirect to total effect (RIT) and the ratio of direct to indirect effect (RID) was used to capture the size of the mediating effect.

**Table 3:** MLE results at the global level and other regions, 2020.

	**All regions**	**Africa**	**Asia**	**Europe & Australia**	**North America**	**South America**
**Observations**	125	40	24	39	13	9
**Panel A: Mediator: Governance index**						
**lgcvddths**	0.421∗∗∗ (0.091)	0.289∗∗ (0.142)	−0.229 (0.191)	−0.152 (0.267)	0.284 (0.389)	−0.733∗ (0.387)
**Constant**	−1.911∗∗∗ (0.454)	−2.467∗∗∗ (0.453)	0.467 (0.834)	2.793∗ (1.621)	−1.492 (2.261)	4.808 (2.492)
**Panel B: Dependent variable: GDP growth rate (%)**						
**Governance**	−0.085 (0.199)	−0.756∗ (0.456)	−0.140 (0.586)	0.059 (0.251)	−0.628 (0.902)	−0.139 (0.699)
**lgcvddths**	−0.812∗∗∗ (0.219)	−0.563 (0.429)	−0.233 (0.564)	−1.178∗∗∗ (0.420)	−0.448 (1.291)	−1.325 (0.961)
**Constant**	−0.234 (01.079)	−1.419 (1.723)	−2.996 (2.408)	3.132 (2.639)	−7.128 (7.479)	2.284 (6.218)
**Panel C: Significance testing of indirect effect**						
**Sobel**	−0.036 (0.084)	−0.219 (0.170)	0.032 (0.137)	−0.009 (0.041)	−0.179 (0.355)	0.102 (0.516)
**Monte Carlo**	−0.029 (0.0842)	−0.200 (0.169)	0.28 (0.191)	0.010 (0.087)	−0.132 (0.468)	0.080 (0.620)
**Panel D: Test for mediation**						
**BK**	X > M∗∗∗ M > Y	X > M∗∗ M > Y∗	X > M M > Y	X > M M > Y	X > M M > Y	X > M∗ M > Y
**ZLC**	X > Y∗∗∗	X > Y	X > Y	X > Y∗∗∗	X > Y	X > Y
**RIT**	0.042	0.280	0.160	0.008	0.285	0.084
**RID**	0.044	0.389	0.138	0.008	0.399	0.077
**Panel E: GOF Tests**						
**LR**$\chi^{2}$**test**	36.460∗∗∗	9.921∗∗	1.584	7.670∗	1.235	5.064
**RMSEA**	0.000	0.000	0.000	0.000	0.000	0.000
**CFI**	1.000	1.000	1.000	1.000	1.000	1.000
**TLI**	1.000	1.000	1.000	1.000	1.000	1.000

∗∗∗, ∗∗, and ∗ denote 1 %, 5 %, and 10 % level of significance respectively. In parenthesis (.) are standard errors. X, M, and Y denote exogenous, mediator, and dependent variables.

**Table 4:** MLE results at the global level and other regions, 2021.

	All regions	Africa	Asia	Europe & Australia	North America	South America
**Observation**	125	40	24	39	13	9
**Panel A: Mediator: Variable-governance**						
**lgcvddths**	0.436∗∗∗ (0.099)	0.485∗∗∗ (0.113)	−0.108 (0.219)	−0.312 (0.210)	0.982∗ (0.544)	−0.332 (1.182)
**Constant**	−2.426∗∗∗ (0.581)	−3.565∗∗∗ (0.492)	0.157 (1.260)	3.931∗∗∗ (1.402)	−6.351∗ (3.573)	2.513 (8.672)
**Panel B: Dependent variable: Economic growth**						
**Governance**	0.298∗ (0.170)	0.999∗∗∗ (0.333)	1.061∗∗ (0.522)	−0.515∗∗ (0.228)	−0.888 (0.705)	0.319 (0.761)
**lgcvddths**	0.459∗∗ (0.202)	0.098 (0.288)	−0.629 (0.564)	0.320 (0.307)	3.873∗∗ (1.548)	2.212 (2.711)
**Constant**	2.823∗∗ (1.178)	5.346∗∗∗ (1.574)	7.363∗∗ (3.226)	5.411∗∗ (2.186)	−17.213∗ (10.130)	−8.819 (19.892)
**Panel C: Significance testing of the indirect effect**						
**Sobel Test**	0.130 (0.080)	0.484∗∗ (0.197)	−0.114 (0.239)	0.161 (0.129)	−0.872 (0.845)	−0.106 (0.454)
**Monte Carlo** **Test**	0.132∗ (0.080)	0.490∗∗ (0.200)	−0.129 (0.274)	0.164 (0.142)	−0.778 (0.857)	−0.115 (1.095)
**Panel D: Test for mediation**						
**BK**	X > M∗∗∗ M > Y∗	X > M∗∗∗ M > Y∗∗∗ X > Y	X > M M > Y∗∗	X > M M > Y∗∗	X > M∗ M > Y	X > M M > Y
**ZLC**	X > Y∗∗∗	X > Y	X > Y	X > Y	X > Y∗∗	X > Y
**RIT**	0.221	0.832	0.154	0.334	0.291	0.050
**RID**	0.283	4.935	0.182	0.501	0.225	0.048
**Panel E: GOF tests**						
**LR**$\chi^{2}$**test**	30.455∗∗∗	27.782∗∗∗	5.504	9.186∗∗	8.036∗∗	0.831
**RMSEA**	0.000	0.000	0.000	0.000	0.000	0.000
**CFI**	1.000	1.000	1.000	1.000	1.000	1.000
**TLI**	1.000	1.000	1.000	1.000	1.000	1.000

∗∗∗, ∗∗, and ∗ denote 1 %, 5 %, and 10 % level of significance respectively. In parenthesis (.) are standard errors. X, M, and Y denote exogenous, mediator, and dependent variables.

**Table 5:** MLE results for disaggregated governance indicators in Africa, 2021.

	Control of corruption	Government effectiveness	Regulatory quality	Rule of law	Voice & accountability	Political stability
**Observations**	40	40	40	40	40	40
**Panel A: Mediator variable: Governance**						
**lgcvddths**	0.177∗∗∗ (0.049)	0.201∗∗∗ (0.051)	0.167∗∗∗ (−0.048)	0.213∗∗∗ (0.046)	0.160∗∗∗ (0.056)	0.249∗∗∗ (0.067)
**Constant**	−1.330∗∗∗ (0.211)	−1.478∗∗∗ (0.219)	−1.318∗∗∗ (0.208)	−1.473∗∗∗ (0.206)	−1.293∗∗∗ (0.245)	−1.774∗∗∗ (0.291)
**Panel B: Dependent variable: Economic growth**						
**Governance**	2.538∗∗∗ (0.759)	2.391∗∗∗ (0.734)	2.214∗∗∗ (0.797)	1.823∗∗ (0.830)	1.136 (0.717)	1.330∗∗ (0.587)
**lgcvddths**	0.133 (0.270)	0.101 (0.277)	0.211 (0.276)	0.194 (0.306)	0.401 (0.281)	0.251 (0.288)
**Constant**	5.165∗∗∗ (1.431)	5.324∗∗∗ (1.488)	4.708∗∗∗ (1.485)	4.473∗∗∗ (1.633)	3.256∗∗ (1.448)	4.149∗∗∗ (1.500)
**Panel C: Significance testing of indirect effect**						
**Sobel Test**	0.450∗∗ (0.183)	0.481∗∗ (0.191)	0.371∗∗ (0.171)	0.389∗∗ (0.197)	0.182 (0.131)	0.331∗ (0.171)
**Monte Carlo** **Test**	0.453∗∗ (0.186)	0.485∗∗ (0.194)	0.375∗∗ (0.174)	0.397∗∗ (0.198)	0.187 (0.135)	0.337∗ (0.174)
**Panel D: Test for mediation**						
**BK**	X > M∗∗∗ M > Y∗∗∗ X > Y	X > M∗∗∗ M > Y∗∗∗ X > Y	X > M∗∗∗ M > Y∗∗∗ X > Y	X > M∗∗∗ M > Y∗∗ X > Y	X > M∗∗∗ M > Y	X > M∗∗∗ M > Y∗∗ X > Y
**ZLC**	X > Y	X > Y	X > Y	X > Y	X > Y	X > Y
**RIT**	0.722	0.826	0.637	0.667	0.312	0.568
**RID**	3.392	4.735	1.756	2.003	0.453	1.317
**Panel E: GOF tests**						
**LR**$\chi^{2}$**test**	25.916∗∗∗	27.346∗∗∗	22.304∗∗∗	25.438∗∗∗	14.333∗∗∗	21.295∗∗∗
**RMSEA**	0.000	0.000	0.000	0.000	0.000	0.000
**CFI**	1.000	1.000	1.000	1.000	1.000	1.000
**TLI**	1.000	1.000	1.000	1.000	1.000	1.000

∗∗∗, ∗∗, and ∗ denote 1 %, 5 %, and 10 % level of significance respectively. In parenthesis (.) are standard errors. X, M, and Y denote exogenous, mediator, and dependent variables.

**Table 6:** MLE results for governance indicators by income level, 2021.

	2020				2021			
Income level	Lower	Lower middle	Upper middle	Upper	Lower	Lower middle	Upper middle	Upper
**Observations**	**16**	**37**	**31**	**40**	**15**	**36**	**33**	**41**
**Panel A: Mediator variable: Governance**								
**Lgcvddths**	0.373∗∗ (0.181)	0.056 (0.114)	−0.144 (0.118)	−0.293∗∗ (0.137)	0.531∗∗∗ (0.153)	−0.060 (0.138)	0.113 (0.135)	−0.431∗∗∗ (0.139)
**Constant**	−2.978∗∗∗ (0.505)	−1.759∗∗∗ (0.451)	0.237 (0.662)	4.311∗∗∗ (0.812)	−3.758∗∗∗ (0.529)	−1.469∗∗ (0.657)	−1.274 (0.918)	5.391∗∗∗ (0.909)
**Panel B: Dependent variable: Economic growth**								
**Governance**	−0.091 (0.709)	−0.717 (0.521)	−0.336 (0.724)	1.553∗∗∗ (0.566)	1.541∗∗ (0.643)	1.667∗∗∗ (0.641)	1.047∗ (0.616)	−0.542 (0.405)
**Lgcvddths**	0.113 (0.577)	−0.781∗∗ (0.361)	−0.301 (0.487)	−0.045 (0.525)	−0.075 (0.512)	0.088 (0.512)	0.220 (0.505)	0.613 (0.401)
**Constant**	−0.540 (2.553)	−0.849 (1.697)	−4.686∗ (2.673)	−8.877 (3.821)	7.014∗∗ (2.751)	6.936∗∗∗ (2.598)	5.294 (3.483)	3.593 (3.215)
**Panel C: Significance testing of indirect effect**								
**Sobel**	−0.034 (0.265)	−0.040 (0.087)	0.048 (0.112)	−0.456∗ (0.270)	0.818∗∗ (0.415)	−0.099 (0.234)	0.119 (0.158)	0.234 (0.190)
**Monte Carlo**	0.010 (0.276)	−0.031 (0.100)	0.047 (0.152)	−0.478∗ (0.286)	0.831∗∗ (0.422)	−0.113 (0.256)	0.118 (0.181)	0.228 (0.206)
**Panel D: Test for mediation**								
**BK**	X > M∗∗M > Y	X > M M > Y	X > M M > Y	X > M∗∗M > Y∗∗∗∗X > Y	X > M∗∗∗M > Y∗∗X > Y	X > M M > Y∗∗∗	X > M M > Y∗	X > M∗∗∗M > Y
**ZLC**	X > Y	X > Y∗∗	X > Y	X > Y	X > Y	X > Y	X > Y	X > Y
**RIT**	0.432	0.049	0.192	0.910	1.01	8.853	0.350	0.276
**RID**	0.302	0.052	0.161	10.076	10.955	1.127	0.538	0.381
**Panel E: GOF tests**								
**LR**$\chi^{2}$**test**	3.826	6.746∗	1.950	12.089∗∗∗	16.206∗∗∗	6.331∗	3.896	15.322∗∗∗
**RMSEA**	0.000	0.000	0.000	0.000	0.000	0.000	0.000	0.000
**CFI**	1.000	1.000	1.000	1.000	1.000	1.000	1.000	1.000
**TLI**	1.000	1.000	1.000	1.000	1.000	1.000	1.000	1.000

∗∗∗, ∗∗, and ∗ denote 1 %, 5 %, and 10 % level of significance respectively. In parenthesis (.) are standard errors. X, M, and Y denote exogenous, mediator, and dependent variables.

#### 4.2.1 Aggregate results

As shown in Table 3, at the global level, a=0.421, b=0.085, and c=0.0812. These are coefficients for the impact of (1) COVID-19 (lgcvddths) on governance (X>M), (2) governance on economic growth (M>Y), and (3) COVID-19 on economic growth (X>Y). While a and c are statistically significant at 1 %, b is insignificant. Given that the impact of governance on the dependent variable is insignificant, the BK mediation condition is not met at the global level. In addition, the Sobel test for the global model is also insignificant. This implies that the difference between c and a∗b (indirect effect) is not statistically significant after controlling for governance. Accordingly, the non-mediation effect of governance is upheld. This result is confirmed by the more robust ZLC test, which relies on the significance of the indirect effect. The ZLC and Monte Carlo statistic of −0.029 is insignificant. Coupled with an insignificant c, the ZLC test suggests that there is direct-only non-mediation.

It can therefore be deduced that there is strong evidence that governance did not mediate the impact of COVID-19 on economic growth at the aggregate level in 2020. For 2021, similar results are documented in which there is no mediation effect for the aggregate sample (see Table 4). Although the aim of the study was to examine the mediating role of governance, an analysis of the impact of COVID-19 on governance and the impact of governance on economic growth was also conducted. As shown in Table 4, at the global level, a = 0.436, b = 0.298, and c = 0.459. While a and c are statistically significant at 1 %, b is insignificant at the standard 5 % level. Given that the mediator impact on the dependent variable is insignificant, the BK mediation condition is unmet.

The results of the COVID-19 economic growth relationship are common in that the impact was negative globally and for every region in 2020. At the global level, c is 0.812 and is significant at 1 %. This entails that in 2020, a country with higher COVID-19 deaths per million, on average, recorded a 0.812 lower economic growth rate. The size is near unitary elasticity, which suggests that COVID-19 had a hugely detrimental effect on growth. For the 125 countries in the sample, the average GDP growth was −3.9 %, while the COVID-19 deaths per million were 346. The results compare very well with [71], who documented that COVID-19 was injurious to global GDP growth. Their study revealed that a daily increase in daily deaths per 100 000 in a quarter led to a 0.85 percentage point slump in GDP growth. The negative impact of COVID-19 on growth is not surprising. Following the outbreak of the COVID-19 pandemic, lockdowns were the standard response. This curtailed economic activity [72], including trade [82], which culminated in a global economic recession [[20], [21], [22], [23]].

Regarding the impact of COVID-19 on governance, the results suggest that governance improved in 2020 and 2021 following the pandemic. The coefficients are 0.421 and 0.436 and significant at 1 %. This suggests that, on average, a country with more deaths per million had better governance by 0.421 and 0.436 units in 2020 and 2021 respectively. The research findings contrast with those of [25], who reported that better governance reduced morbidity and mortality after adjusting for non-governance variables in a negative binomial model. These results can be explained by the irony that more developed countries, often with better governance, were the most affected ones.

In some cases, this can reflect how lockdowns benefitted governance. Because many spheres of life were restricted, the usual channels through which governments interacted with citizens, such as corruption, the rule of law, voice, and accountability, were constrained. Lockdowns were instituted during the COVID-19 pandemic to contain the spread of the virus and to protect public health. Even though lockdowns inevitably restricted individual liberties, they played a significant role in preventing the abuse of state power and promoting citizen voice in multiple ways [83]. These ways included, but are not limited to, (1) decision-making transparency, (2) consultation with citizens, (3) regular updates and feedback mechanisms, and (4) engagement with civil society.

For instance, governments openly communicated the rationale behind closure measures, the data underlying their decisions, and the anticipated outcomes [83]. Transparent communication built trust between states and their citizens and enabled the latter to comprehend the necessity of restrictive measures. Some governments also solicited input from citizens, experts, and other stakeholders in developing closure measures. This approach gave citizens a say in formulating policies that directly affected their lives, which enhanced their sense of ownership and reduced feelings of helplessness.

The GOF tests (Panel E) for all the results tables show that at the global level, across all regions, and for all six measures of governance in Africa (see Table 4), and across all the income levels, the RMSEA p-value was zero, which is well below the threshold of 0.05. This entails that there is no specification error in the SEMs. The CFI and TLI are also all equal to 1. This depicts that the hypothesised model is fitted relatively better than the baseline model, resembling a perfect fit. In a few instances, the LR $\chi^{2}$ was significant, which seemingly suggests poor model fit. However, given the criticism raised on the methodology, the GOF tests were based on the RMSEA, CFI, and TLI. The tests confirmed with confidence that the SEMs were correctly specified.

#### 4.2.2 Regional level results

To control for heterogeneity, the study regressed the SEM on the five regions for 2020 and 2021. Across all regions in 2020, the results from the BK, Sobel, and ZLC tests showed that the null hypothesis of no mediation cannot be rejected at the standard 5 % significance level. Accordingly, governance did not play any mediating role in the impact of COVID-19 on economic growth. Moving to 2021, the results revealed that only in Africa did governance play a significant mediating role. The BK conditions were fulfilled as *a* = 0.485 and *b* = 0.998 were statistically significant at 1 %, while *c* = 0.098 was statistically insignificant. This shows evidence of complete mediation. The Sobel test echoed this finding. The p-value of 0.014 means that the null hypothesis of non-mediation was rejected at a 5 % level of significance. It follows that the inclusion of governance in the model rendered the impact of COVID-19 on economic growth insignificant.

Using the ZLC approach, the indirect coefficient of 0.49 was statistically significant at 5 %. This confirms the indirect-only (full) mediation effect of governance in Africa in 2021. The RIT and RID for Africa were 0.832 and 4.935. The two measure the magnitude of the mediation effect, with lower values indicating low mediation effects. For the former, this entails that approximately 83 % of the effect of COVID-19 was mediated by governance. For the latter, it can be inferred that the mediated effect was approximately 4.9 times as large as the direct effect of COVID-19 on growth. Compared with the 2020 RIT (28 %) and RID (0.389), it is clear that governance’s moderating effect was more pronounced in 2021 than in 2020. For other regions, the RIT and RID values were low. However, it is essential to note that for the aggregate sample, Europe and North America, the RIT and RID increased between 2020 and 2021. Even though the mediation was statistically insignificant, the increase in the size is a notable result.

The study’s headline finding is that the mediation effect of governance was supported only in Africa in the second year of the COVID-19 pandemic. The findings are, at a glance, unexpected, especially given Africa’s poor governance, inferior health infrastructure, and high poverty rates. Against expectations, Africa was the least affected region regarding COVID-19 mortality. In 2020 and 2021, the sampled African countries had the lowest average COVID-19 deaths per million of just 51 and 231 against the aggregate sample averages of 347 and 740. At the same time, Africa’s economic growth was the least affected. In 2020, the sampled African countries recorded an average growth rate of −1.7 %. The aggregate sample registered a growth rate of −3.9 %. In North America, the economy shrunk by −9.7 %. From this, it is deduced that COVID-19 was more injurious to more developed economies. Several reasons have been put forward to explain this anomaly.

According to Ref. [84], Africa benefitted from hotter, less windy weather conditions, a younger population, and better herd immunity from repeated disease exposure. This provided an opportunity for governance systems in Africa, despite being inferior to other regions, to better mediate the impact of COVID-19 on growth. Without a doubt, the enforcement of lockdown measures involved abuse of state power by governments, which often led to human rights abuse [85] and corruption in, for example, the procurement of personal protective equipment [86,87], and political instability [88]. However, the results suggest that governance benefitted from the pandemic. In Africa, most governance violations involved government agencies against the people. With free movement and business as usual, clashes between the government and the people were frequent. However, freedom of movement violations hid people from the government; hence fewer altercations and better governance. For instance Ref. [88], posited that the lockdowns saw civil society and democracy actors migrate to digital democracy engagement platforms such as Zoom, Twitter, etc. As such, contact with state actors was reduced; hence better governance. Furthermore, the use of e-government, such as in South Africa, allowed the use of social media platforms as e-governance tools to generate and share epidemic intelligence [89]. This went a long way in reducing the information costs of fighting the pandemic, as predicted by the institutional economics theory [8]. The pandemic, to some extent, also united the people and their governments. In South Africa [90], argued that the government and citizens entered into an informal and undeclared social contract for a common cause over time. In a report on the impact of the COVID-19 pandemic on governance, peace, and security in the Sahel [41], noted a moderate impact. These reflections cement the argument that, to some extent, governance benefitted from the pandemic, allowing it to play a moderating role in economic recovery in Africa.

The existence of mediation in 2021 and not in 2020 shows the lag effect of governance response to the pandemic. In 2020, the pandemic came as a huge shock. Existing legal frameworks were not compatible with the needed lockdown enforcement. Countries had to craft new laws and go through mandatory bureaucratic procedures. This limited governance’s mediating role. However, by 2021, most governance systems were in place, which translated to increased mediating roles. The specific measures implemented varied significantly based on the outbreak’s severity, the capacity of healthcare systems, and each country’s cultural and political circumstances. Although legal frameworks differed across countries, central laws were formulated and implemented in response to COVID-19. These were in the following sectors and social activities: public health emergency declarations, quarantine and isolation, stay-at-home orders, border control and travel restrictions, social distancing, physical distancing, face mask mandates, and personal protective equipment requirements.

Since a mediating role in Africa is documented, the study extended the analysis by examining the impact of six governance indicators. The results are presented in Table 5.

The results in Table 5 show that the magnitude and significance of mediation varied with the form of governance indicators. The results confirm full mediation evidence for control of corruption, government effectiveness, regulatory quality, and the rule of law. Both the Sobel test and the ZLC and Monte Carlo tests were statistically significant at 5 % for these governance components. Of these governance indicators, government effectiveness had the most significant mediation effect. The RIT (0.826) and RID (4.735) denote that approximately 83 % of the effect of COVID-19 on economic growth was mediated by better governance effectiveness, while the mediated effect was around 4.7 times as large as the direct effect of COVID-19 on economic growth. There is evidence of COVID-19 spread mitigation driven by governance effectiveness across the world. New Zealand and South Korea took early and decisive action to restrict travelling and implement lockdowns and instituted aggressive testing and contact tracing, which reduced the spread of the virus [91]. In Japan, Germany, and Taiwan, clear communication of pandemic information in the form of frequent updates and consistent messaging helped governments to build public trust [92]. Other acts of government effectiveness were seen through timely enhancement of healthcare infrastructure in the UKand Italy, instantaneous rollout of COVID-19 vaccination programmes, and socio-economic support to the vulnerable and poor in countries such as Canada, the USA, and South Africa [92]. See Appendix 4 for more examples of governance related interventions in specific countries.

Political stability, absence of violence, and voice and accountability did not play any significant mediating role. For political stability and absence of violence, both a and b were significant, with c being insignificant. According to the BK approach, this shows partial mediation. However, the Sobel, ZLC, and Monte Carlo tests were insignificant, which confirmed no-effect non-meditation.

The finding here can be important for policymaking against future economic shocks. The four mediating governance indicators are more important in economic recovery from shocks. Thus, in periods of shock, governments must focus more on controlling corruption and promoting good government effectiveness and the rule of law.

#### 4.2.3 Income-level results

Further to regional groupings, the mediation analysis was disaggregated according to income levels. The results are presented in Table 6.

The results in Table 6 show that in 2020, there was no evidence of mediation across all the income groupings, at least according to the ZLC criteria. The Monte Carlo indirect effect was not statistically significant at the standard 5 % level. However, going by the BK approach, there was partial mediation in the upper-income group. For the group, a and b were significant, while c was not. However, the Sobel test was insignificant at the 5 % level. Hence, the BK detected partial mediation.

Nonetheless, the ZLC approach was preferred, which rejected any mediation role for governance. Turning to 2021, the results point to governance mediation for low-income countries only. For these countries, a = 0.531 and b = 1.541 were statistically significant, while c = -0.075 was not. This was complemented by significant Sobel and Monte Carlo indirect effect statistics of 0.818 and 0.831 respectively. It follows that governance had an indirect-only (full) mediation effect on the impact of COVID-19 on economic growth in low-income countries in 2021.

The striking observation from the disaggregated income-level analysis is that the results mirrored those of the regional analysis in two ways. Firstly, the time lag effect was also present. Secondly, the presence and absence of governance mediation correlated with countries’ development levels. As the income levels increased, governance’s mediation effect on economic recovery fizzled out. As shown in Table 4, mediation was detected only in Africa in 2021. The region had the lowest income per capita of USD 406.45. The common trend is that low-income countries are associated with poor governments, as in Africa. Accordingly, the results resembled those in Africa, as discussed previously.

## 5 Conclusion

This section concludes the study by summarising the research objective, methodology, and key findings. It also provides the policy recommendations and ends by highlighting the study’s weaknesses and areas of further research.

### 5.1 Summary of the study

The main objective of the study was to examine whether, how, and to what extent governance mediated economic recovery after the COVID-19 epidemic. The specific objective was to assess the extent to which the mediation role varied with regions and levels of income. A priori, better-governed countries should be more prepared, respond better, and have better insulation against shocks. On the contrary, more developed and better-governed regions and countries experienced more severe COVID-19 cases and economic recession. In questioning the role of governance in economic recovery from COVID-19, the study makes two contributions to the literature. Firstly, to the best of our knowledge, this is the first study to bring together governance, COVID-19, and economic growth relationships. Secondly, the study brought global evidence and disaggregated the relationships according to regions and income levels. To achieve this, the study followed a multi-stage econometric analysis.

At first, the principal component analysis approach was used to construct a composite governance index from the World Bank’s six Worldwide Governance Indicators. After that, mediation based on PLS-SEM estimation was employed for analysis. Data drawn from 125 countries were used for the global model. In line with the study’s objective, estimation was then categorised by regions as follows: Africa (40), Asia (24), Europe and Australia (39), North America (13), and South America (9). Further disaggregation by income level included lower (16), lower middle (37), upper middle (31), and upper (40). The headline finding was that governance did not mediate at the aggregate level in 2020 and 2021. However, regional analysis revealed a full mediation effect in Africa and for low-income countries in 2021.

Further disaggregating governance by indicators in Africa revealed complete mediation for control of corruption, government effectiveness, regulatory quality, and the rule of law. Government effectiveness had the most significant mediating effect, while political stability, absence of violence, and voice accountability did not play any significant mediating role. In the interest of these findings, essential policy recommendations are proffered.

### 5.2 Policy recommendations

It can be concluded that effectively navigating global crises and achieving sustainable economic recovery requires strengthening governance structures, promoting transparency, accountability, and responsiveness, and encouraging international cooperation, especially for African and low-income economies. Broadly, investments in strengthening governance structures, enhancing institutional capacities, and enhancing decision-making processes will be crucial for improved pandemic preparedness and response in the future. The following specific recommendations are suggested.

#### 5.2.1 Internationalisation of governance systems

There is a need to internationalise governance through international cooperation and collaboration to reap synergy yields. Accordingly, the establishment of an international governance institution in the spirit of UN-driven frameworks is suggested. This will allow governments to trade best practices, coordinate efforts, and exchange resources to ensure a more unified and effective response to future natural, health, and economic challenges.

#### 5.2.2 Multi-stakeholder, cross-sector collaborations

International efforts should be complemented by national, multi-agency support, and cross-sector collaborations for rapid responses. This will increase the resilience of countries to shocks. Other countries can learn from the USA’s Federal Emergency Management Agency. It uses a community-based approach that maximises the synergies of private and non-profit sectors (businesses, disability organisations, faith-based organisations, etc.) and in conjunction with the involvement of central, local, and tribal governmental partners [93]. Such arrangements can build trust relationships that minimise contradictions and confusion during a crisis. Multi-stakeholder, cross-sector governance frameworks should prioritise reducing mistrust, which was a major issue during the fight against COVID-19. This can be achieved by designing communication mechanisms led by trusted voices, building demographically sensitive awareness of shocks, and creating space for stakeholders to express themselves.

#### 5.2.3 Innovation and digitalisation of governance systems

Furthermore, governance systems need to be more innovative. For instance, governments should invest in e-governance tools (social media, artificial intelligence, etc.) while managing their potential disruptive tendencies (there was a great deal of misinformation on COVID-19 causes, treatment, vaccinations, etc.). Digital platforms can also be used to enhance transparency through improving public access to data regarding public expenditure, procurement procedures, and allocations of recovery funds. This would mitigate corruption and bolster confidence in the recovery process. Moreover, it promotes transparent budgeting procedures that enable citizen involvement in decision making concerning resource distribution, particularly about recovery money.

#### 5.2.4 Enacting anti-corruption strategies

Anti-corruption agencies should be enhanced by granting them the authority and resources to investigate and prosecute corruption associated with crisis (COVID-19) recovery initiatives. This encompasses transparency in public procurement and contracts associated with recovery initiatives. Governments must guarantee that the utilisation of money is consistently reported to the public, encompassing comprehensive details on fund allocation and resultant outcomes, thereby fostering a culture of accountability.

#### 5.2.5 Promoting economic diversification and resilience

In the context of economic recovery, governance frameworks must provide focused assistance for sectors significantly affected by shocks (COVID-19), including tourism, retail, and small enterprises. This assistance should also promote economic diversification to establish long-term resilience. Furthermore, governments should advocate for recovery policies that emphasise sustainability, including investments in renewable energy, green infrastructure, and innovation. Governance frameworks ought to prioritise long-term economic stability over transient solutions.

### 5.3 Further research opportunities

This study provided some novel evidence on the mediating role of governance in economic recovery; however, it is not exhaustive. While the study emphasised the mediating role of governance, there are other factors that could play the same role but were not covered in the analysis. These factors include the quality of healthcare, human capital productivity, and economic parameters such as monetary and fiscal policy initiatives. Future studies can therefore provide improved evidence by combining and comparing the mediating roles of governance in these factors.

## CRediT authorship contribution statement

**Regret Sunge:** Writing – review & editing, Writing – original draft, Visualization, Validation, Software, Methodology, Formal analysis, Data curation, Conceptualization. **Calvin Mudzingiri:** Writing – review & editing, Supervision, Software, Resources, Project administration, Investigation, Funding acquisition, Formal analysis, Data curation, Conceptualization. **Nkosingiphile Mkhize:** Writing – review & editing, Visualization, Validation, Project administration, Investigation, Conceptualization.

## Data availability

The data that support the findings of this study are available from the corresponding author, Regret Sunge, upon reasonable request.

## Declaration of generative artificial intelligence and artificial intelligence-assisted technologies in the writing process

During the preparation of this work, the authors used Grammarly in order to improve language and readability, with caution. After using this tool/service, the authors reviewed and edited the content as needed and take full responsibility for the content of the publication.

## Declaration of competing interest

The authors declare that they have no known competing financial interests or personal relationships that could have appeared to influence the work reported in this paper.

## Figures and Tables

**Table:** 

**Number**	**Africa**	**Europe**	**Asia**	**North America**	**South America**	**Total**
1	Algeria	Albania	Azerbaijan	Bahamas	Argentina	
2	Angola	Austria	Bangladesh	Barbados	Bolivia	
3	Benin	Belarus	Bhutan	Belize	Brazil	
4	Botswana	Belgium	China	Canada	Chile	
5	Burundi	Bulgaria	India	Cuba	Colombia	
6	Cameroon	Croatia	Indonesia	El Salvador	Costa Rica	
7	Central African Republic	Cyprus	Iran	Guatemala	Ecuador	
8	Chad	Denmark	Iraq	Haiti	Paraguay	
9	Congo	Estonia	Israel	Honduras	Uruguay	
10	Cote d'Ivoire	Finland	Japan	Jamaica		
11	Democratic Republic of Congo	Georgia	Jordan	Mexico		
12	Egypt	Germany	Kazakhstan	Panama		
13	Eswatini	Greece	Lebanon	United States		
14	Ethiopia	Hungary	Malaysia			
15	Gabon	Iceland	Mongolia			
16	Gambia	Ireland	Myanmar			
17	Ghana	Italy	Oman			
18	Guinea	Kosovo	Pakistan			
19	Kenya	Latvia	Philippines			
20	Madagascar	Lithuania	Singapore			
21	Mali	Luxembourg	South Korea			
22	Mauritania	Moldova	Sri Lanka			
23	Mauritius	Netherlands	Thailand			
24	Morocco	New Zealand	Uzbekistan			
25	Namibia	Nicaragua				
26	Niger	Norway				
27	Nigeria	Portugal				
28	Rwanda	Romania				
29	Saudi Arabia	Russia				
30	Senegal	Serbia				
31	Sierra Leone	Slovakia				
32	Somalia	Slovenia				
33	South Africa	Spain				
34	Sudan	Sweden				
35	Tanzania	Switzerland				
36	Togo	Turkey				
37	Tunisia	Ukraine				
38	Uganda	United Kingdom				
39	Zambia	Australia				
40	Zimbabwe					

**Total**	**40**	**39**	**24**	**13**	**9**	**125**

**Table A2:** 1 Governance indicators correlation matrix.

Variable	Corruption		Government Effectiveness		Political Violence		Regulatory Quality		Rule of Law		Voice & Accountability	
	2020	2021	2020	2021	2020	2021	2020	2021	2020	2021	2020	2021
**Corruption**	1	1										
**Governance Indicators**	0.928	0.931	1	1								
**Political Violence**	0.827	0.807	0.806	0.795	1	1						
**Regulatory Quality**	0.906	0.913	0.955	0.949	0.808	0.797	1	1				
**Rule of Law**	0.948	0.951	0.951	0.954	0.828	0.820	0.946	0.950	1	1		
**Voice and Accountability**	0.815	0.814	0.791	0.782	0.796	0.788	0.824	0.822	0.816	0.828	1	1

**Table A2.2:** Principal Components

Component	Eigenvalue		Difference		Proportion		Cumulative	
	2020	2021	2020	2021	2020	2021	2020	2021
Comp1	5.3214	5.3079	5.0263	4.9999	0.8869	0.8847	0.8869	0.8847
Comp2	0.2951	0.3080	0.0884	0.0938	0.0492	0.0513	0.9361	0.9360
Comp3	0.2067	0.2142	0.1099	0.1247	0.0344	0.0357	0.9705	0.9717
Comp4	0.0967	0.0895	0.0531	0.0458	0.0161	0.0149	0.9866	0.9866
Comp5	0.0437	0.0437	0.0072	0.0071	0.0073	0.0073	0.9939	0.9939
Comp6	0.0364	0.0367	.	.	0.0061	0.0061	1.0000	1.0000

**Fig. A2:**
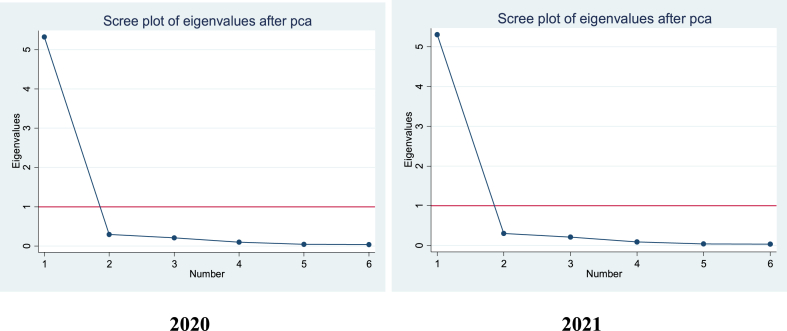
1 Scree Plots

Source: Authors’ computation from PCA estimates

**Fig. A2.2:**
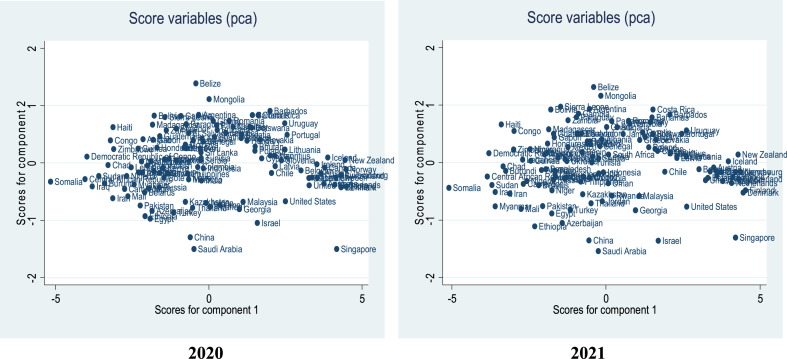
Principal Component Analysis Score Variables

Source: Authors’ computation from PCA estimates

**Figure:**
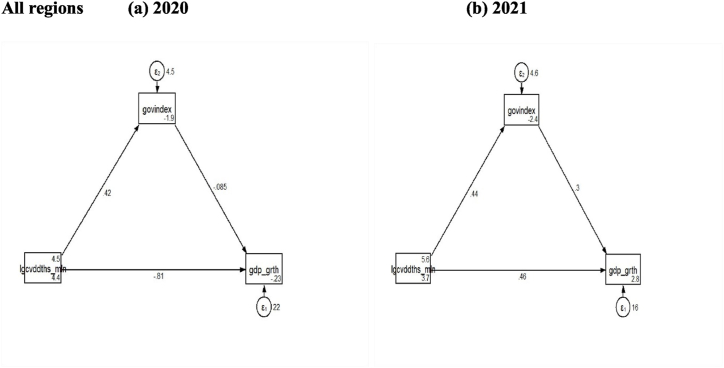


**Figure:**
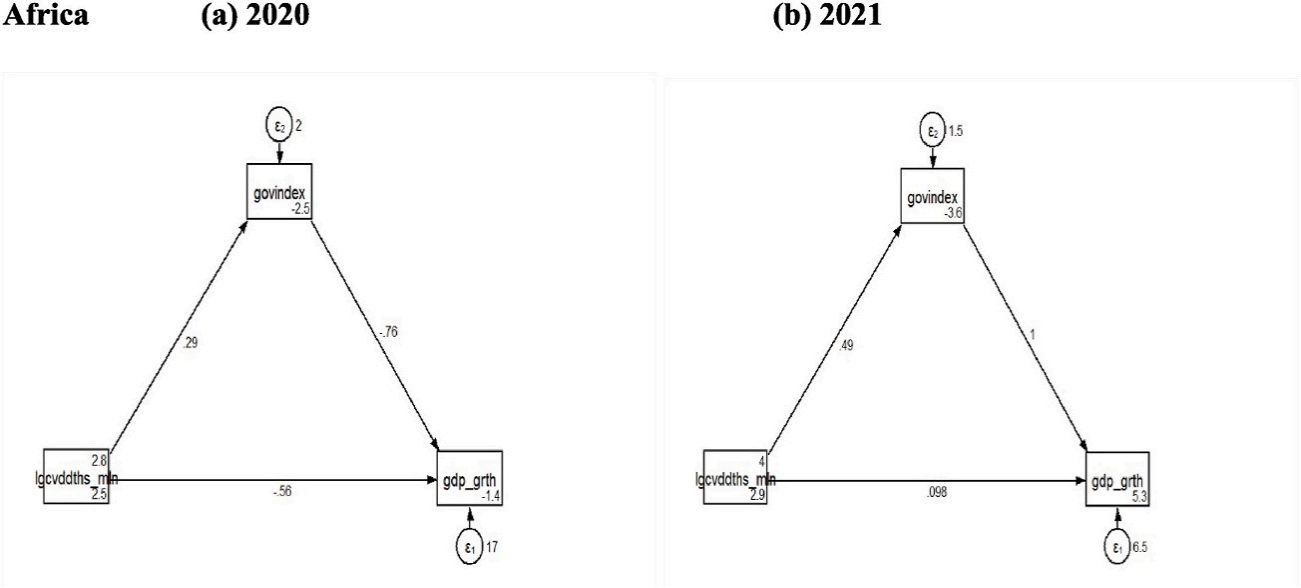


**Figure:**
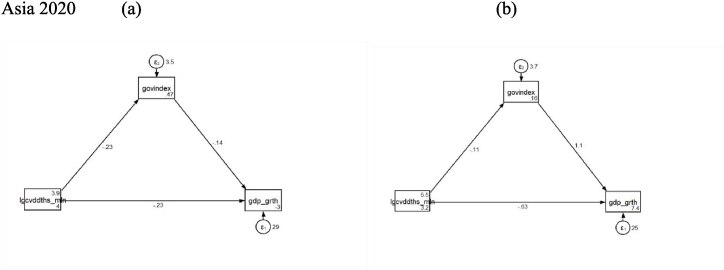


**Figure:**
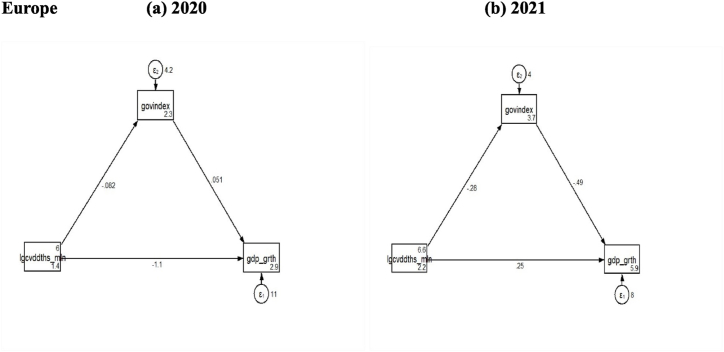


**Figure:**
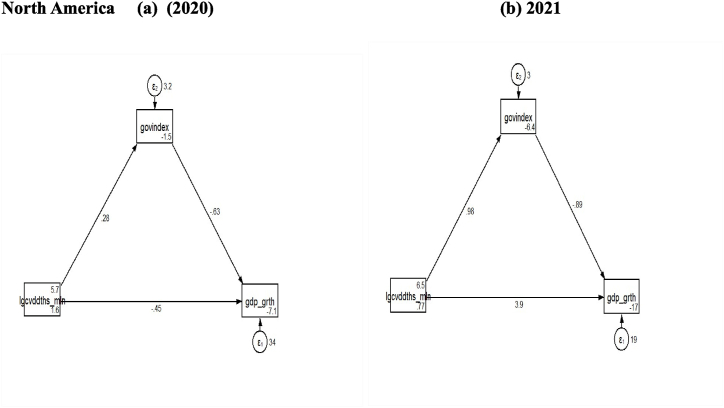


**Figure:**
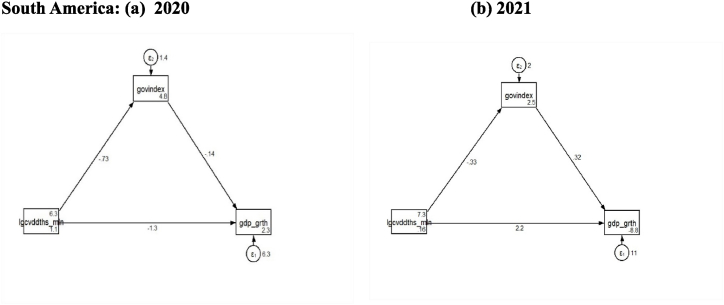


**Figure:**
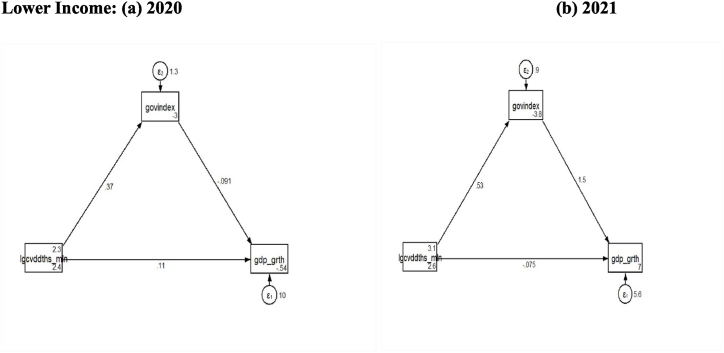


**Figure:**
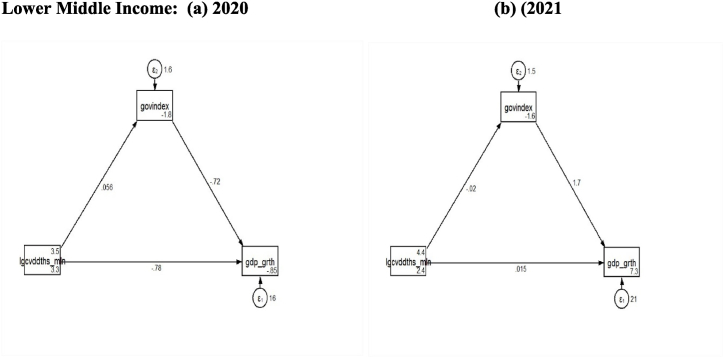


**Figure:**
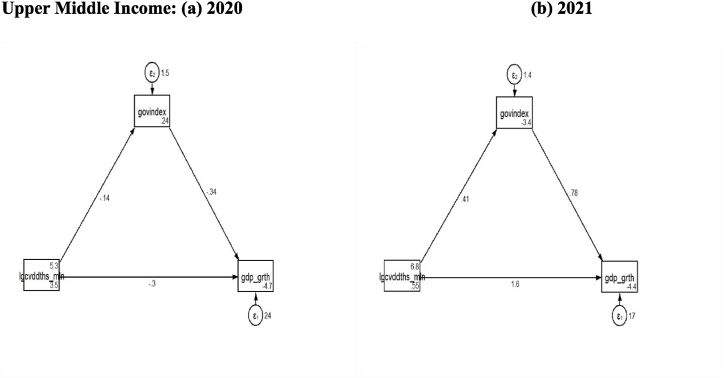


**Figure:**
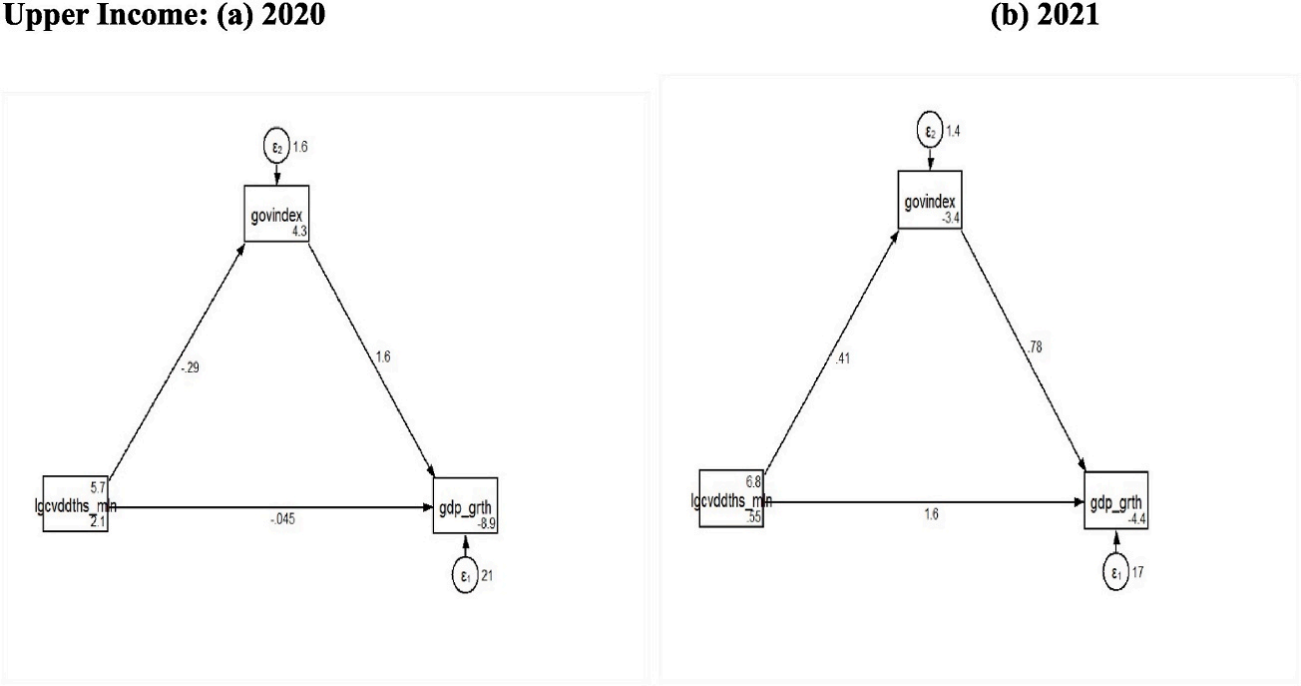


**Table:** 

**Country**	**Description of case(s)**	**Governance practice**
Germany	Germany's response to COVID-19 was marked by compliance with the rule of law via the Infection Protection Act, which authorised federal and state governments to implement legal measures to address the pandemic. Legal power facilitated the prompt implementation of lockdowns, mask regulations, and travel limitations. The judiciary established checks and balances, guaranteeing that restrictive actions were proportional and consistent with constitutional rights. This example illustrates how a strong legal framework that harmonises public health and civil liberties can enhance public trust and compliance, which are essential components of an effective crisis response.	Rule of Law
New Zealand	The government of New Zealand, under Prime Minister Jacinda Ardern, demonstrated accountability through transparent decision-making and consistent public communications about COVID-19 policy. Daily briefings conducted by officials, including health specialists, who elucidated decisions and assumed accountability for results. Explicit performance milestones, including the reduction of transmission rates, were tracked and publicly reported. New Zealand's methodology underscores the critical role of accountability in cultivating confidence and affirming the legitimacy of governmental activities, particularly in periods of crisis.	Accountability – Leadership in crisis
South Korea	South Korea's COVID-19 response was predicated on transparency, with the government disseminating real-time information regarding the virus's transmission and the efficacy of control efforts. A government website that is updated daily with information on new cases, testing rates, and regions impacted by outbreaks. Utilisation of smartphone notifications to apprise citizens of potential exposures and health directives. Transparency fostered public trust and collaboration, demonstrating that access to credible information is essential in crisis management. This substantiates the claim that transparent communication channels enhance governance results.	Transparency
Taiwan	The Taiwanese government demonstrated response by implementing early and decisive measures, such as stringent border restrictions and comprehensive testing, largely influenced by its experience with the SARS epidemic. A swift reaction facilitated by the prompt activation of the Central Epidemic Command Centre (CECC) within days of the initial reported case. Continuous revisions and modifications to policy informed by emerging facts and global events. Taiwan's achievement exemplifies the significance of an adaptive governance framework, especially during the initial phases of a crisis. The capacity to respond swiftly to shifting information illustrates the necessity for governing institutions to be flexible in the face of changing conditions.	Responsiveness
Norway	Norway’s COVID-19 response implemented stringent procedures to guarantee transparency and accountability in procuring health resources, thereby reducing corruption risks. An online platform facilitated public oversight of government contracts about pandemic responses, guaranteeing equitable and transparent resource distribution. The anti-corruption agency of Norway diligently scrutinised fund utilisation to prevent the emergence of misuse during emergency responses. The emphasis on anti-corruption initiatives in Norway's administration underscores the significance of oversight during crises. This comparative example underscores that upholding transparency and accountability, especially in challenging circumstances, mitigates inefficiencies and unscrupulous practices in the allocation of emergency resources.	Corruption and anticorruption
Vietnam	The government of Vietnam throughout the epidemic was characterised by significant public engagement, especially through community-based health efforts and widespread adherence to regulations. Collaboration with local leaders and health volunteers who performed door-to-door health assessments, increased awareness, and distributed masks. Community-engaged public message programs aimed at curbing viral transmission. Vietnam's grassroots methodology demonstrates that governance frameworks including public involvement can enhance societal responses to crises. This case illustrates that including citizens in governance procedures improves collective response during public health emergencies.	Public participation

## Appendix 2 Principal Component Analysis

Principal Component Analysis (PCA) was used to generate the composite governance index. The PCA converts several possibly correlated variables into principal components, a smaller number of uncorrelated variables. The six governance indicators are strongly correlated, with all correlations at least 0.8, as shown in Table A2.1. Hence including all of them will cause multicollinearity problems.

PCA identifies the underlying components responsible for variation in a data [1]. The component/indicator with the highest variation is the most important and is assigned a higher weight. The principal components is symbolised as the sum of all indicator variables, denoted by x, weighted by principal component-specific loadings, L:

(1) $${PC}_{pc}=\sum_{i=1}^{N}L_{i}x_{i}\ldots \ldots \ldots \ldots \ldots \text{.}$$

The weights are then assigned as follows:

(2) $$W_{i}=\left\langle \sum \left|L_{ij}\right|E_{j}\right\rangle \ldots \ldots \ldots \ldots \ldots \text{.}$$

Where W is the assigned weight, E is the eigen value, i = 1,2 …,n indicators, j = 1, 2, … … are the principal components. The weighted index is therefore given as:

(3) $$I_{ih}=\frac{\sum X_{i}W_{i}}{\sum W_{i}}\ldots \ldots \ldots \ldots \ldots \text{.}$$

The weights given to the six indicators are given in Table A2.2. In both years, component 1 (Control of corruption) had the highest variation and gets the highest proportion of 0.8869 (2020) and 0.8847 (2021). This means that the first component represents 89 % of the information in the sample. The governance index was created from the first component, since it contributed more than 16 % of the variation (100/6).

### PCA Scree Plots and Score Variables

The use of the first component was confirmed by the scree plots of eigen values as shown in Figure A2.1. The plot is based on Kaiser’s rule in which only components with eigen values greater than greater than 1 should be retained. In Table … component 1 has eigenvalue values of 5.32 (2020) and 5.31 (2021). This means that the first component account for at least as much variation as in any of the original variables.

The score variables locate the countries in the component space and are shown in Figure A2.2. The plots show that Somalia had low loadings in both components while Singapore has high loadings in component 1 and low in component 2.

## Appendix 3 SEM Path Diagrams

The graphs show the path diagrams for the SEM results for the respective region and years. The path statistics are the same as those reported in Table 3, Table 4, Table 5, Table 6.

## Footnotes

3 See Appendix 2 for an explanation of the methodology.

## Footnotes

2 Authors’ illustration based on [69].

## Footnotes

1 These are: (1) control of corruption, (2) government effectiveness, (3) voice and accountability, (4) political stability and lack of violence, (5) regulatory quality, and (6) rule of law. The indicators range from −2.5 (worst) to 2.5 (best).

4 SEM path diagrams are shown in Appendix 3.
